# Effect of Accelerated Weathering on the Thermal, Tensile, and Morphological Characteristics of Polypropylene/Date Nanofiller Composites

**DOI:** 10.3390/ma15176053

**Published:** 2022-09-01

**Authors:** Basheer A. Alshammari, Othman Y. Alothman, Abdullah Alhamidi, Mohammad Jawaid, Hamid M. Shaikh

**Affiliations:** 1 Materials Science Research Institute, King Abdulaziz City for Science and Technology, Riyadh 11442, Saudi Arabia; 2 Department of Chemical Engineering, College of Engineering, King Saud University, Riyadh 11421, Saudi Arabia; 3 Department of Biocomposite Technology, Institute of Tropical Forestry and Forest Products (INTROP), University Putra Malaysia, Serdang 43400, Malaysia; 4 SABIC Polymer Research Centre, Department of Chemical Engineering, King Saud University, Riyadh 11421, Saudi Arabia

**Keywords:** date palm, nanofillers, accelerated weathering, polypropylene, thermal properties, tensile strength

## Abstract

The aging of polypropylene (PP) composites reinforced with date palm nanofiber (DNF) was investigated in this study in order to predict their long-term performance. To produce composites, date palm nanofibers in the range of 1–5 wt% loading were dry-melt-blended with polypropylene. These biocomposites were then subjected to UV exposure (Xenon arch source) for accelerated weathering for 250 and 500 h according to a standard method. The change in thermal properties before and after accelerated weathering was investigated using thermogravimetric analysis (TGA) and differential scanning calorimetry (DSC). TGA analysis shows that the maximum degradation temperature for sample at 1 wt% loading was 382.7 °C, which slightly decreased to 379.9 °C after 250 h and to 367.7 °C after 500 h of weathering. DSC analysis also revealed lower crystallinity of the same samples after exposure to accelerated weathering. Mechanical properties were also studied to identify the damage induced by accelerated weathering. The tensile strength of the highest loading (5 wt%) of the sample was found to occur at 34.83 MPa, which was slightly lowered to 31.64 after 500 h treatment. A minimal decrease in tensile strength, deterioration, and weathering-induced oxidation indicates the excellent stability of these composites. Therefore, our study provides insight into the aging behavior of such composites, which may be useful in dry conditions, as well as nonstructural automotive and other parts for which minimum tensile strength (~25 MPa) is specified.

## 1. Introduction

Wood polymer composites (WPCs) are considered attractive and promising materials due to their superior efficiency, low moisture absorption ability, and endurance against ecological impact, such as fungal and insect invasion. Considerable research has been conducted to explore the multifunctional properties of such composite materials [1,2]. Composite materials are usually produced from two or more constituent materials with dissimilar chemical and physical properties. Biocomposite materials are a type of composite material composed of environmentally friendly lignocellulosic additives, which are reinforced with a polymer matrix. WPCs are particularly suitable as a substitute for both natural and synthetic wood in applications in which wood comes into direct contact with a humid environment, such as decking, household goods, flooring, and fencing. Furthermore, WPCs are widely used in the construction sector for the manufacture of various household articles, office appliances, and furniture [3,4,5,6,7]. 

Because they are derived from waste, there has been continuous interest in developing industrial and consumer products that incorporate both plastic and natural fillers. The primary incentive has come from several objectives, including a reduction in material cost and the development of products with specific properties that can be recycled [8]. The ability of biocomposite materials to tolerate stress is determined by their bonding ability. The compatibility of natural materials with polymer matrices is widely established to play a key role in determining the qualities of final composite products. As a result, a variety of chemical and thermal treatments have been used to increase the compatibility of composite component materials [9,10,11].

Many researchers have been intrigued by the weatherability qualities of WPC as an industrial material. Wood, on the other hand, is a biological material that is decomposed by degradation of organic factors, including fungi, insects, and bacteria [12]. Wood is also hygroscopic in nature, which allows it to absorb moisture in wet media or desorb moisture in a dry medium by evaporating the water outside the cell [13]. As a result of this tendency, wood can become a dimensionally unstable structure. Therefore, in several exterior applications, the sturdiness and dimensional stability of wood is a critical factor in the final performance of WPC products [14]. 

Several polymer matrices including polyolefins are used to fabricate biocomposite by utilizing natural and/or synthetic wood as a reinforcing material. Owing to its various advantages, including cost and mechanical qualities, PP is among the most extensively used thermoplastics. However, PP also has underprivileged weathering qualities and is vulnerable to heat, light, and oxidation, limiting its potential for long-term industrial applications. In order to develop composites based on PP, various accelerated weathering studies have been conducted. These artificial weathering tests mimic the effects of irradiance, temperature, humidity, water, and light on materials exposed to the elements. Shimizu et al. [15] and Lv et al. [16] plotted the weatherability of PP using both laboratory and outdoor exposure tests. Based on the accelerated laboratory aging data, they found that outside weathering behaviors could be anticipated satisfactorily, allowing for a quick assessment of the service endurance of polymer matrices in a short amount of time, as opposed to the arduous, expensive, and time-consuming nature of true outside exposure trials.

The durability and weathering characteristics of PP/natural filler composite materials have been thoroughly evaluated. For instance, Aydemir et al. [14] found that heat-treated beechwood-reinforced PP composites accelerated weathering and decay resistance. They discovered that heat treating wood improves the antifungal effectiveness of composites. Finally, heat-treated wood offers more application potential than untreated wood composites. However, in a weathering environment, heat-treated wood tends to grey out quickly, which could be a disadvantage for typical outdoor use.

Josef et al. [17] studied the UV degradation of short sisal fiber/PP composite materials with treated using UV radiation and water. They found that tensile strength decreases with longer irradiation time. Photo oxidation and chain session were revealed to be the causes of degradation. Tensile characteristics were lowered as a result of this phenomenon. The authors concluded that treatment using urethane derivatives provided good interfacial adhesion. Tian et al. [18] investigated the accelerated weathering properties of a wood flour/PP composite in depth. After UV exposure, an investigation of mechanical properties showed that virgin PP had the worst weathering properties when compared to its composites. Similar investigations on the characterization and acceleration of weathering of synthetic wood/PP composites have been published elsewhere [19,20,21,22]. Abu-Sharkh et al. [8] investigated the degradation effect of date palm fiber/PP composites under both natural and artificial weathering conditions in Saudi Arabia. They discovered date palm fiber/PP composites were more resistant than unfilled PP in both natural and accelerated weathering environments. 

In view of our objective, few studies have investigated natural-fiber-reinforced PP composite materials. Therefore, the goal of this research is to investigate the accelerated weathering performance of PP composites reinforced with date palm nanofibers (DNFs) obtained in previous studies [23] with various DNF loadings to determine/improve thermal, mechanical, and morphological properties for outdoor applications as an alternative to wood materials using the least amount of DNF filler possible. Thus, the exploration and characterizations of non-metallic composites based on nanofillers derived from third-generation agro-waste (lignocellulosic waste) biomass is a novel aspect of this work. This will not only help to effectively reduce moisture absorption ability but also help to maintain the mechanical strength of these composites, which can be useful in outdoor applications in arid environments. 

## 2. Materials and Methods

### 2.1. Establishment of Nanofillers

**Figure 1:**
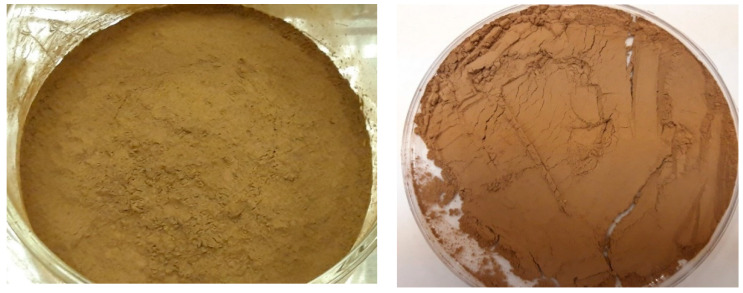
Digital images of date palm nanofiller (DNF).

Nanofillers were obtained by adopting our previously described method [23]. Dry milling with a planetary ball mill (Pulverisette 7 Premium, Fritsch Co., Idar-Oberstein, Germany) was used to obtain fine powder. Milling was performed in a 15 mL zirconia container with 10 mm zirconia balls at a rate of 300 RPM throughout the process. The average size of these fillers was reported to be in the dimeter range of 30–50 nm, with a length range of 1–10 mm. Digital images of the filler are presented in Figure 1. 

### 2.2. Preparation of PP/DBF Biocomposites

Biocomposites were made with homopolymer PP obtained from the National Industrialization Company (TASNEE, Riyadh Province, Saudi Arabia). The homopolymer has a melt flow index (MFI) of 3 g/10 min and a density of 0.9 g/cm^3^ and is commercialized under the brand name PP3030. Melt mixing followed by injection moulding were used to create the PP/DNF composite samples. The DNF was blended with PP at a ratio of 1–5 wt%, and the resulting mixture was fed into a melt compounder (DSM Xplore micro-compounder, 15 cm^3^, Sittard, The Netherlands). The composite melt-mixing process was carried out in speed-controlled mode at a melting temperature of 200 °C, with a screw speed of 100 rpm and a mixing period of 5 min. When the specified mixing period had passed, the molten mass was collected in a warmed collector using a cylindrical piston assembly. The collector temperature was fixed at 200 °C, which was the same as the mixing temperature. The injection-molded standard specimen (ISO-527 type IBA) was prepared using a microinjection molder (DSM Xplore, Sittard, the Netherlands) for tensile testing. These specimens are termed PP/DNF-1, PP/DNF-2, PP/DNF-3, PP/DNF-4, and PP/DNF-5, where the number reflects the nanofiller’s loading percentage. None of these composites was prepared using a weak ester-bond-containing compatibilizer, such as maleic-anhydride-grafted polypropylene (MAPP). Table 1 presents information about the formulation of the composites.

### 2.3. Accelerated Weathering Test

UV-accelerated weathering was performed on tensile specimens as per ASTM G155 standard using a UV chamber (Atlas Ci5000 Weather-Ometer, Mount Prospect, IL, USA). The samples were exposed to 250 and 500 h of UV light and water spray. The detail condition are as follow: cycles: 1; filter: daylight; irradiance: 0.35 W/m^2^; wavelength: 340 nm; 102 min UV ray exposure; black-panel temperature: 63 °C;18 min light and water spray. These samples were designated as 250PP/DNF-1, 250PP/DNF-2, 250PP/DNF-3, 250PP/DNF-4, 250PP/DNF-5, 500PP/DNF-1, 500PP/DNF-2, 500PP/DNF-3, 500PP/DNF-4, and 500PP/DNF-5.

### 2.4. Thermal Analysis

To assess thermal stability, a TGA analysis was performed before and after UV exposure using a Shimadzu thermal analyzer (Model: DTG-60H, Tokyo, Japan). Exactly 15 mg of the sample was placed in the alumina pan, and the samples were heated at a rate of 20 °C/min from room temperature to 600 °C. The assessment was conducted in a nitrogen atmosphere with an air velocity of 50 cm^3^/min, and the resulting weight loss was noted. Three measurements were carried out on each sample, and mean values were reported. Differential scanning calorimetry (DSC, Shimadzu DSC-60, Tokyo, Japan) was used to investigate melting, crystallization temperature, and heat flow. The samples were heated from room temperature to 250 °C at a rate of 10 °C/min, holding samples for 3 min at 250 °C before they were cooled to 25 °C at the same rate. At least four heating and cooling cycles were performed on each sample, with the first cycle being omitted to remove the samples’ thermal history. The mean value of the following three cycles was then reported. Equation (1) was used to calculate the degree of crystallinity (Xc):

(1) $$X_{c}\left(\%\right)=\frac{{\Delta H}_{m}}{(1-{\Phi )\Delta H}_{0}}\times 100$$

where ΔHm is the calculated enthalpy of melting for the composite; Φ is the amount of filler; and ΔH_0_ is the theoretical enthalpy of melting for 100% crystalline PP, which is reported to be 209 J/g [24].

### 2.5. FTIR Analysis

Fourier transform infrared spectroscopy (ATR-FTIR, Thermo Scientific, Winsford, UK) analysis was performed using a Nicolet iN10 FTIR microscope with a germanium microtip. The analysis was performed in the wavenumber range of 650–4000 at a resolution of 4 cm^−1^ with 64 scans. Three spectral measurements were performed on each sample in various locations to analyze the functional groups on the surface of the composites.

### 2.6. Tensile Strength Analysis

The ISO-527 standard specimen (Type 1BA) was used, and tests were carried out in uniaxial tension mode. The tensile properties of the composite samples were determined using a Hounsfield H100 KS series tensile testing machine (Salfords, UK). At least five measurements were taken for each specimen, and the mean values were reported.

### 2.7. Surface Morphological Analysis

To detect the surface morphology, polarized microscopic observations (POMs) were realized in transmission mode. Tensile specimen samples before and after weathering treatments were used as such on an Olympus microscope equipped with a digital camera (BX51 system microscope, Tokyo, Japan). Similarly, scanning electron microscopy (SEM) was used to analyze the morphological characterizations (JEOL, JSM-6360A, Tokyo, Japan). A cryofacture surface of the samples was applied to conductive carbon tape attached to the sampling stub. Prior to examination, all samples were gold-sputtered.

## 3. Results and Discussion

### 3.1. Thermal Analysis

**Figure 2:**
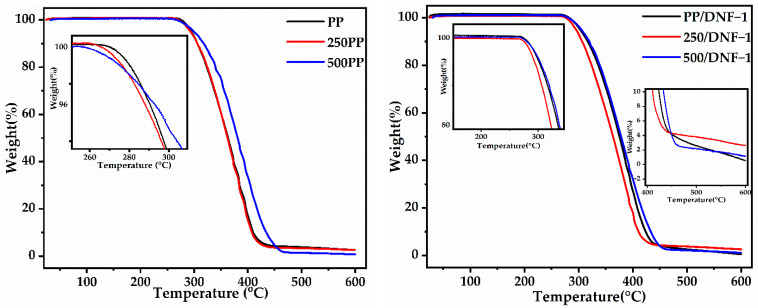

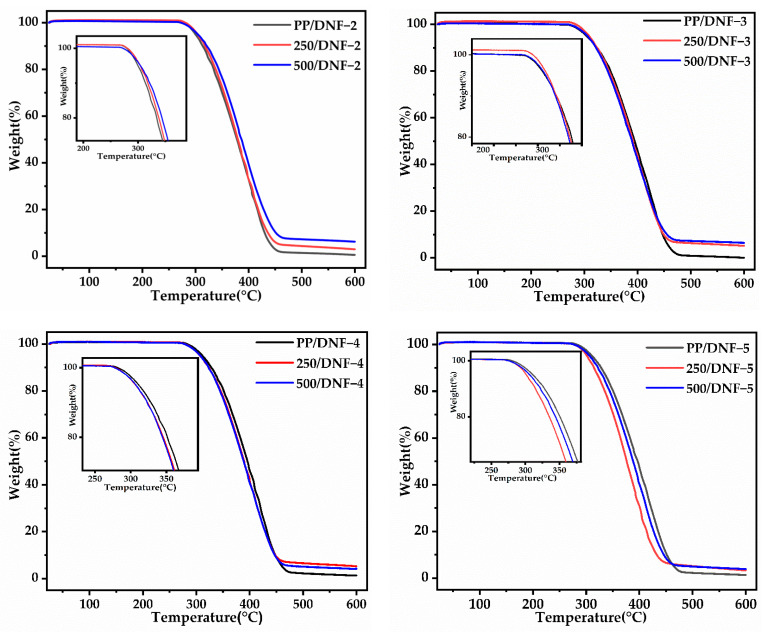
TGA graph of samples before and after 250 h and 500 h weathering.

Thermogravimetric data (TG) of the samples are presented in Figure 2. A one-step decomposition process was observed an all samples. The temperature range between 300 and 500 °C corresponds to first stage due to decomposition of the PP/DNF composites. The temperatures interrelated to onset, weight loss of 5% (T_5_), and maximum degradation temperature are presented in Table 2. In general, the starting decomposition temperature (T_onset_) and maximum degradation temperature in composites increases with the addition of a filler. Whereas composites with DNF loading indicate a starting decomposition temperature of ~280 °C, neat PP tends to start to decompose at a temperature of approximately 266 °C. This indicates that DNF enhances thermal stability by effectively blocking a molecular thermal motion in the composites, as well as the stabilization of the nano fillers [25,26]. Furthermore, the loading percentage of the filler and exposure time for weathering causes a slight decrease in the decomposition temperature. However, this could be a result of the heat-sink effect from the residual ash, as well as the thermal insulation effect provided by the large quantity of polymer matrix [27]. Therefore, such composites are suitable for use in outdoor applications. Furthermore, increased residual content at 600 °C suggests that this filler is less combustible than neat PP.

**Figure 3:**
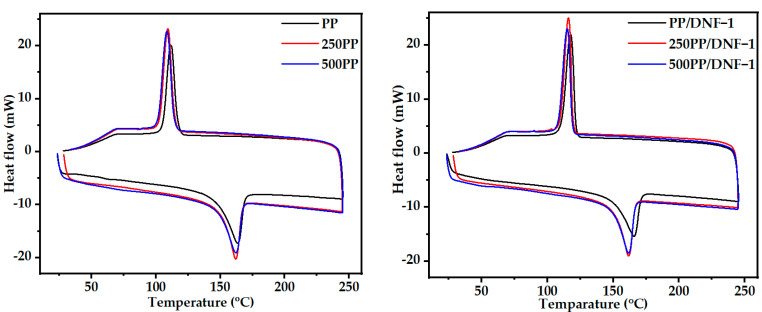

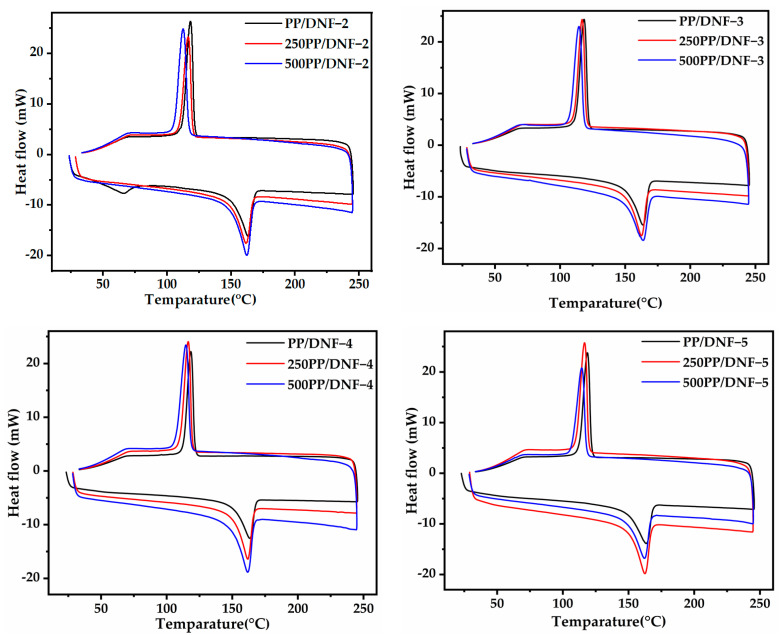
DSC thermogram of samples before and after weathering.

Similarly, the dynamic thermograms (heating and cooling) of the neat PP matrix and the PP/DNF composites are shown in Figure 3. Table 3 compiles the non-isothermal parameters of melting and crystallization. The melting temperatures (Tm) of the PP/DNF composite were found to be slightly lower, whereas the crystallization temperature (Tc) was found to be higher than that of the clean PP. Moreover, the addition of filler particles had more influence on Tc, but overall crystallinity is was decreased compared to that of the neat PP, which might be attributed to the fillers’ poor or non-existent nucleating ability. The decrease in crystallinity caused by a large amount of fillers may hinder the mobility of the polymer chain, causing crystal development to be slowed [28]. Similarly, the increase in enthalpy of crystallization (ΔHc) in composites also indicates imperfect crystallization. These rigid filler particles may occupy the interstitial position of the polymer chain, preventing orderly rearrangement of the polymer chain. The decrease in enthalpy of melting also suggests a reduction in the mobility of the polymer chain. In general, the PP crystallinity decreased from 49% to 29% in concert with the increase in the filler amount, as well as with accelerated weathering. In the case of accelerated weather exposure, samples with less exposure time showed slightly higher crystallinity, possibly due to the filler particles acting as nucleating agents, facilitating heterogeneous PP nucleation and improving crystallinity. However, the prolonged exposure condition may have prevented PP spherulite from growing in all directions, resulting in a slight decrease in crystallinity. Similar effects on crystallinity characteristics dependent on filler amount have been observed in other PP-based composite systems [29].

### 3.2. Functional Group Analysis

**Figure 4:**
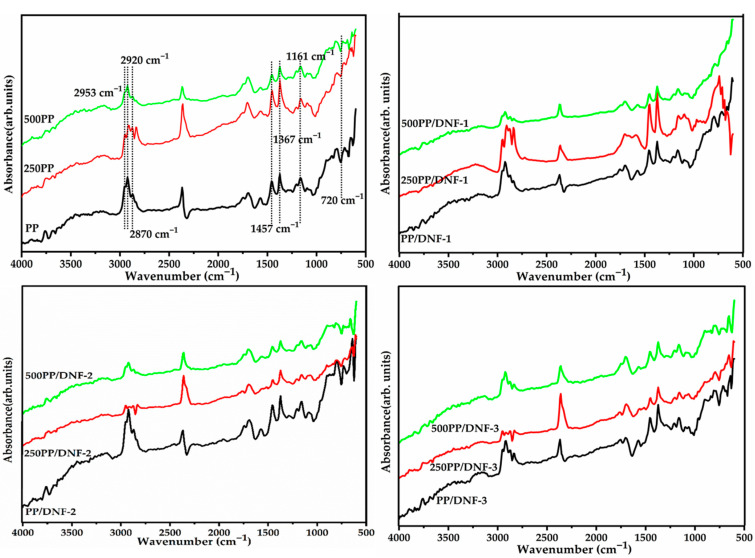

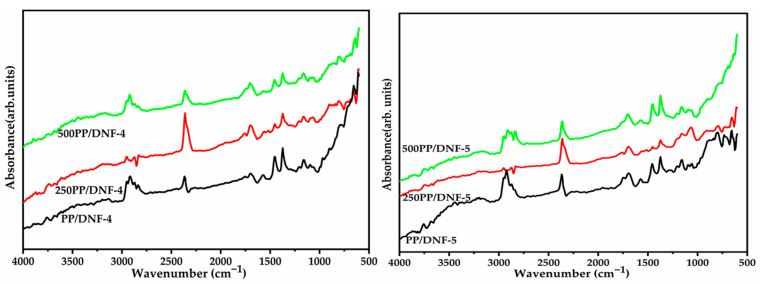
FTIR spectra of PP/DNF composite samples before and after 250 h and 500 h weathering.

The changes in composition of the filler functionalities with processing, as well as with accelerated weathering, were analyzed by FTIR analysis and are exemplified in representative Figure 4. The filler’s functional groups preserved their intensity according to the FTIR analysis. Due to the clear dominance of the matrix, the FTIR of composites generally exhibits characteristic peaks of pristine PP. The strong peak at 3000–2800 cm^−1^ is caused by the C–H stretching vibrations of PP chains. The bending vibrations of –CH_3_ and –CH_2_ are ascribed to the peaks at 1367 cm^−1^ and 1457 cm^−1^, respectively. Furthermore, minor peaks at 1161 cm^−1^, 998 cm^−1^, and 720 cm^−1^ attributed to –CH_3_ symmetric deformation and –CH_3_ rocking vibrations [30]. The presence of aliphatic C-H stretching of cellulosic components is suggested by the FTIR peaks for DNF in the composites with very low intensity that overlapped with the PP at 2850 cm^−1^ and 2920 cm^−1^. In addition, the peak at 1450 cm^−1^ can be attributed to CH_2_ symmetric vibration, which is attached to C6 of cellulose, whereas the peaks at 1160 cm^−1^ and 1050 cm^−1^ are caused by C–O–C at the glycosidic and –C–O–C– at the cellulose backbone, respectively [31], indicating that the composites contain a significant amount of lignocellulosic filler. Even after the specified weathering conditions, the intensity of the carbonyl peak did not appear to be very intense, indicating that the filler and matrix were not oxidized to an extensive degree.

### 3.3. Mechanical Properties

**Figure 5:**
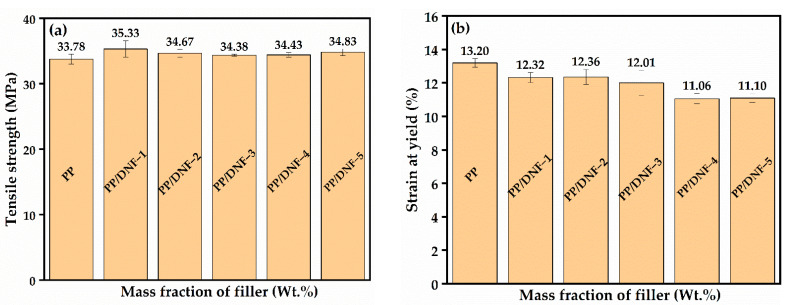

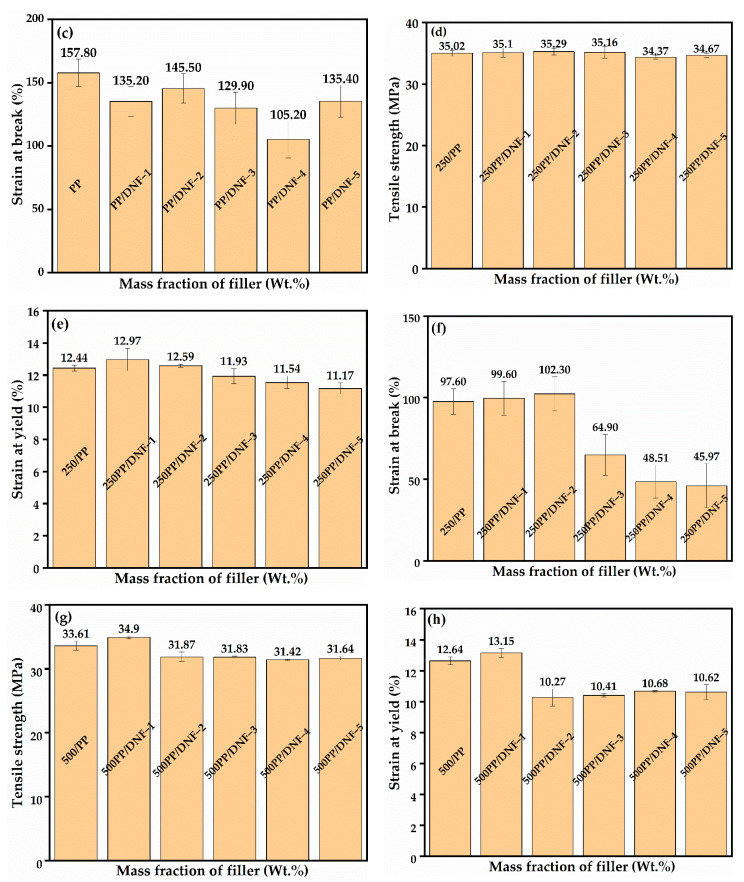

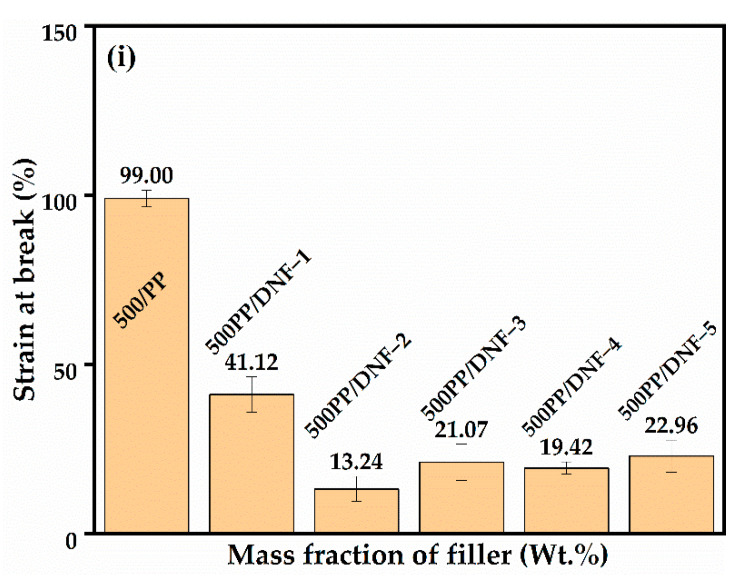
Mechanical properties of composite samples. (**a**) Tensile strength of PP/DNF samples; (**b**) strain at yield (%) of PP/DNF; (**c**) strain at break (%) of PP/DNF; (**d**) tensile strength of 250PP/DNF samples; (**e**) strain at yield (%) of 250PP/DNF; (**f**) strain at break (%) of 250PP/DNF; (**g**) tensile strength of 5000PP/DNF samples; (**h**) strain at yield (%) of 500PP/DNF; (**i**) strain at break (%) of 500PP/DNF samples.

Figure 5 depicts the mechanical properties of samples before and after weathering, including tensile strength, strain at yield, and strain at break. With the addition of the filler, as well as when subjected to accelerated weathering, the tensile strength values remain unchanged. The weak interfacial adhesion between the fillers and the matrix could be to blame for this phenomenon. These composites were made without the assistance of a compatibilizer; however, with the addition of a suitable compatibilizer, the tensile strength can be increased. Strain at yield and strain at break, on the other hand, decreased in the composites, which can be attributed to the stiffer nature of the fillers and the considerable differences in the coefficient of thermal expansion between the matrix and the filler. Similar properties in other PP-based composite system have been observed [32].

### 3.4. Surface Appearance and Morphological Properties

**Figure 6:**
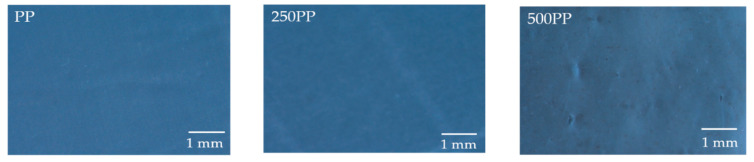

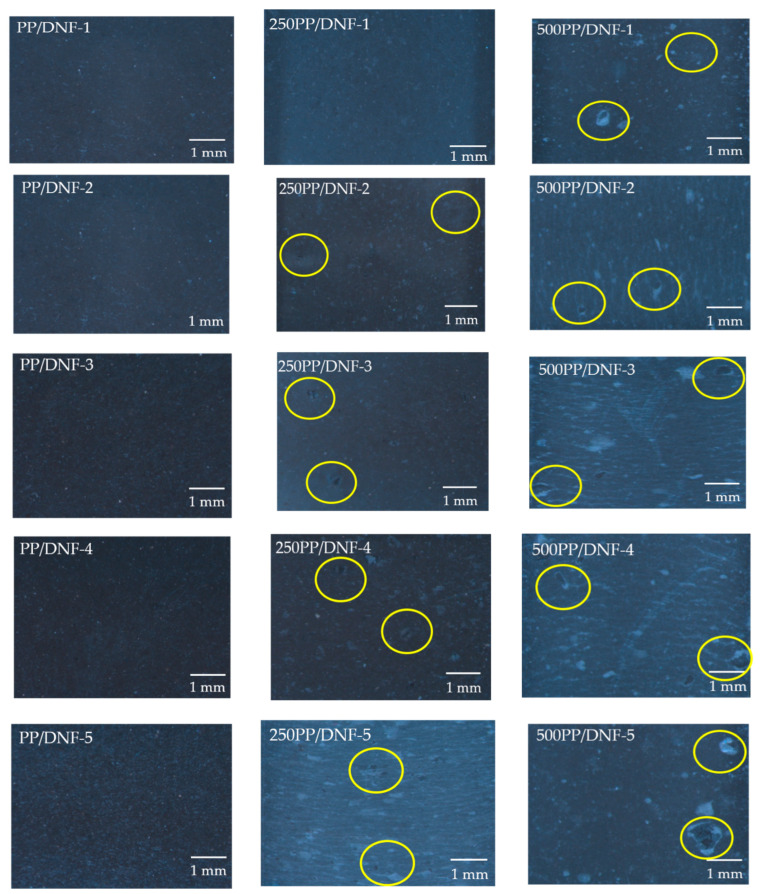
Microscopic surface observations by polarized microscopy (POM).

**Figure 7:**
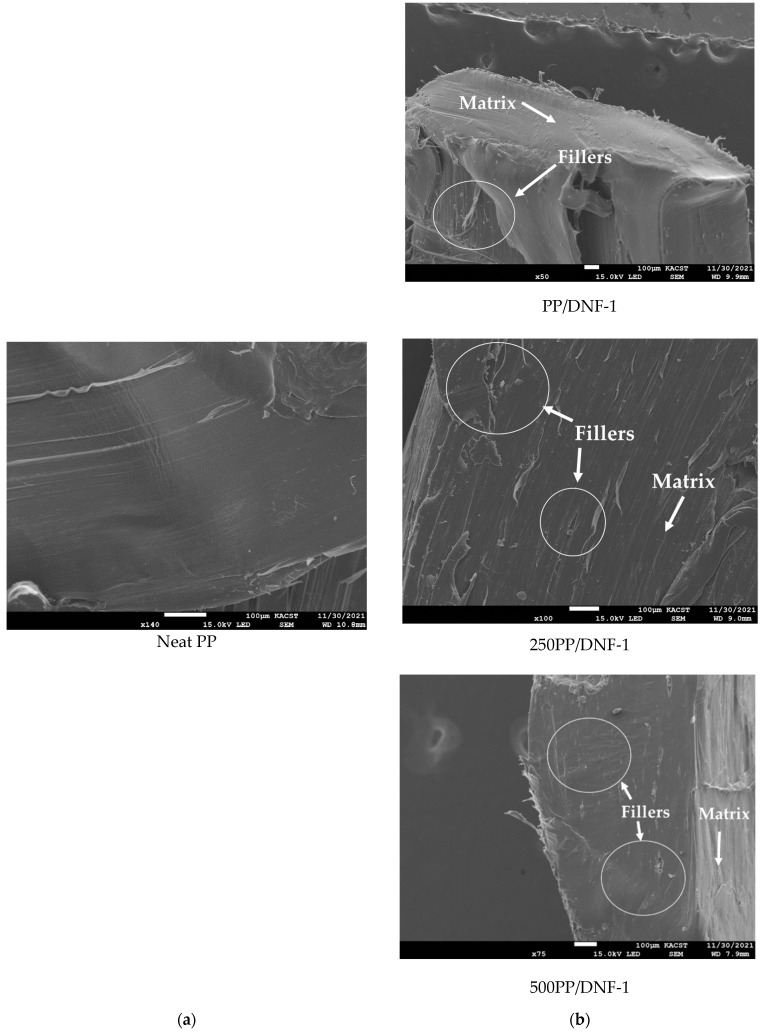
Representative SEM images of the cryo-fracture surface of (**a**) PP, (**b**) PP/DNF-1, 250PP/DNF-1, and 500PP/DNF-1.

Figure 6 shows microphotographs of the surface appearance of the composite samples both unexposed and exposed to weathering at two time intervals. In all the samples, uniform distribution of the fillers can be clearly observed. The surface of unexposed samples appears to be very smooth, whereas exposed samples have some tiny voids (indicated by the yellow circle in some images). With increased exposure time, these voids and tiny cracks become more visible in the studied area due to light discoloration around them. This effect is caused by small cracks in the polymeric matrix surrounding the filler as a result of cyclic dilatation during weathering [33]. Furthermore, lignocellulosic filler swells in response to humidity, increasing stress at the filler–matrix interface and leading to surface cracking. This type of tiny crack is commonly observed in UV-treated PP surfaces as a result of polymer chain scission caused by UV irradiation [34]. Overall, the lack of significant major cracks and deep voids implies that these nanofillers inhibit photodegradation of the PP matrix. Nonetheless, scanning electron microscopy (SEM) was also used to examine the surface (cryo-fractured) of neat PP and composites, and images are shown in Figure 7. The morphological structure is considerably influenced by the nature of the fillers, matrix, and their contact. The filler is dispersed uniformly in the matrix, and no pullout effect is observed. No filler pullout effect is observed in the accelerated weathered samples. However, a ductile fracture surface is clearly observed as a result of the weathering treatments.

## 4. Conclusions

The goal of this study was to learn more about the aging behavior of polypropylene reinforced with nanofillers derived from waste date fiber for use in arid environments. These composites were prepared without the use of a weak ester-bond-containing compatibilizer, such as maleic anhydride grafted polypropylene. Even after 250 and 500 h of accelerated weathering, the thermal stability of these composites remained remarkably constant. The crystallization behavior of the composites was lower than that of neat PP, possibly owing to the low or non-nucleating ability of date palm nanofillers. Similarly, the tensile strength of the composites was unaffected by weathering, although strain at yield and break showed a decreasing trend due to the ductile behavior of the composites. No filler pull-out occurred during processing or weathering treatments, according to the morphological analysis. The lack of oxidation of the filler and fibers during weathering indicates that such composites are suitable for outdoor application in arid environments.

## Figures and Tables

**Table 1:** List of PP/DNF composites produced in the present study.

Sample ID	Composition of DNF (wt%)
Neat PP	0% DNF (0) + PP (100)
PP/DNF-1	1% DNF (1) + PP (99)
PP/DNF-2	2% DNF (2) + PP (98)
PP/DNF-3	3% DNF (3) + PP (97)
PP/DNF-4	4% DNF (4) + PP (96)
PP/DNF-5	5% DNF (5) + PP (95)

**Table 2:** Thermal characteristic of the composite samples.

Samples	T_onset_	T_5W%._	DTG (T_max_)	Residue (W%)
PP	266.47 (±0.60)	285.76 (±1.15)	368.47 (±0.8)	0.55 (±0.05)
PP/DNF-1	282.50 (±0.80)	299.88 (±1.61)	388.59 (±1.49)	0.93 (±0.25)
PP/DNF-2	283.65 (±1.17)	302.98 (±1.84)	395.08 (±1.08)	1.93 (±0.25)
PP/DNF-3	284.21 (±0.30)	304.55 (±0.92)	410.95 (±1.57)	2.70 (±0.72)
PP/DNF-4	285.13 (±0.86)	311.42 (±1.28)	415.85 (±2.24)	3.20 (±0.30)
PP/DNF-5	287.20 (±1.71)	317.16 (±1.74)	418.55 (±0.67)	3.50 (±0.87)
250PP	270.64 (±0.75)	293.53 (±0.60)	350.26 (±1.20)	0.75 (±0.20)
250PP/DNF-1	278.90 (±1.40)	295.45 (±1.26)	377.43 (±0.71)	1.20 (±0.47)
250PP/DNF-2	281.48 (±0.71)	304.57 (±1.58)	387.76 (±2.14)	1.84 (±0.85)
250PP/DNF-3	283.87 (±0.35)	308.73 (±1.32)	392.08 (±1.72)	2.76 (±0.55)
250PP/DNF-4	286.01 (±0.57)	305.41 (±1.02)	402.51 (±1.95)	3.83 (±0.70)
250PP/DNF-5	291.31 (±0.71)	300.34 (±0.71)	412.71 (±2.71)	4.23 (±0.35)
500PP	268.16 (±0.45)	298.14 (±1.73)	348.18 (±0.50)	0.77 (±0.31)
500PP/DNF-1	279.83 (±1.51)	304.23 (±1.01)	379.58 (±0.54)	0.85 (±0.31)
500PP/DNF-2	275.75 (±0.39)	307.57 (±0.87)	367.46 (±1.30)	2.46 (±1.13)
500PP/DNF-3	270.62 (±0.41)	309.65 (±0.60)	365.49 (±1.83)	2.93 (±1.71)
500PP/DNF-4	272.06 (±1.19)	310.11 (±1.95)	363.48 (±2.28)	3.36 (±2.31)
500PP/DNF-5	268.60 (±1.90)	312.06 (±1.03)	354.68 (±3.75)	4.23 (±1.45)

**Table 3:** DSC characteristics of the composite samples.

Sample	T_m_ °C	ΔH_m_ (J/g)	Tc °C	ΔH_c_ (J/g)	X_c_ (%)
PP	163.48 (±1.13)	103.15 (±3.20)	111.51 (±0.05)	99.42 (±2.75)	49.35 (±1.00)
PP/DNF-1	163.85 (±1.25)	68.01 (±4.50)	117.60 (±0.90)	91.37 (±3.20)	32.87 (±1.2)
PP/DNF-2	163.47 (±1.33)	65.30 (±5.40)	118.15 (±1.25)	89.64 (±5.35)	31.88 (±1.4)
PP/DNF-3	163.69 (±0.88)	64.59 (±1.00)	118.46 (±1.12)	87.13 (±4.10)	31.86 (±0.95)
PP/DNF-4	163.24 (±1.44)	64.21 (±2.50)	118.41 (±1.35)	81.85 (±4.55)	32.00 (±1.33)
PP/DNF-5	163.37 (±1.51)	58.04 (±5.90)	118.47 (±1.07)	80.84 (±5.60)	29.23 (±1.21)
250PP	161.89 (±1.26)	73.33 (±2.15)	109.16 (±2.15)	95.68 (±1.26)	35.09 (±2.25)
250PP/DNF-1	161.58 (±1.35)	73.17 (±1.65)	115.05 (±2.80)	92.08 (±3.90)	35.36 (±1.09)
250PP/DNF-2	161.71 (±2.01)	70.16 (±4.45)	116.48 (±1.10)	89.91 (±3.23)	34.25 (±3.21)
250PP/DNF-3	162.57 (±2.22)	69.77 (±3.70)	116.89 (±0.50)	87.25 (±5.30)	34.42 (±2.20)
250PP/DNF-4	161.77 (±1.52)	67.44 (±2.25)	116.31 (±0.75)	86.26 (±3.55)	33.61 (±1.44)
250PP/DNF-5	162.45 (±2.33)	67.07 (±3.30)	116.43 (±1.00)	84.31 (±2.45)	33.78 (±2.50)
500PP	162.08 (±0.50)	74.82 (±1.20)	108.47 (±0.09)	92.88 (±1.50)	35.80 (±1.33)
500PP/DNF-1	161.68 (±1.21)	71.24 (±3.35)	116.10 (±0.98)	88.96 (±3.44)	34.43 (±1.45)
500PP/DNF-2	162.19 (±1.15)	70.85 (±1.30)	112.43 (±1.42)	86.54 (±4.45)	34.59 (±2.11)
500PP/DNF-3	161.86 (±1.81)	69.54 (±4.50)	114.51 (±1.30)	82.50 (±3.42)	34.30 (±1.58)
500PP/DNF-4	161.76 (±1.54)	64.25 (±2.20)	114.66 (±1.28)	81.11 (±2.22)	33.02 (±2.70)
500PP/DNF-5	162.05 (±2.91)	63.57 (±1.80)	114.37 (±1.64)	78.90 (±5.57)	31.75 (±3.45)

## Data Availability

Data are contained within the article.
